# *Yersinia enterocolitica* Prosthetic Joint Septic Arthritis Successfully Treated with Ceftriaxone

**DOI:** 10.1155/2021/5547577

**Published:** 2021-12-10

**Authors:** Hafez M. Abdullah, Mansi Oberoi, Abdelmohaymin Abdalla, Smitha Narayana Gowda, Moataz Ellithi

**Affiliations:** Department of Internal Medicine, University of South Dakota Sanford School of Medicine, Sioux Falls, SD, USA

## Abstract

*Yersinia enterocolitica* is a Gram-negative coccobacillus that is known to cause gastroenteritis and symptoms mimicking appendicitis or terminal ileitis. It is also one of the culprit infections implicated in causing reactive arthritis. Rarely, it can cause musculoskeletal infections including osteomyelitis, septic arthritis, and discitis. We describe the case of a 70-year-old female with multiple comorbidities who presented with left knee pain and swelling after recent gastroenteritis. She was found to have *Yersinia enterocolitica* septic arthritis in her left knee prosthetic joint. The patient underwent an exchange of her prosthetic material and was successfully treated with a six-week course of ceftriaxone. Our article aims to highlight a rare manifestation of *Yersinia enterocolitica* infection and to point out an important differential for reactive arthritis after *Yersinia enterocolitica* infection.

## 1. Introduction

*Yersinia* enterocolitica is a Gram-negative coccobacillus, and pigs are the primary reservoir [1]. It has many serotypes, the most common serotypes being *Y. enterocolitica* with O:3 and O:9 [1]. It primarily causes gastroenteritis and symptoms mimicking appendicitis or terminal ileitis [1]. It is also known to be a cause of postinfectious reactive arthritis. Rarely, it can cause musculoskeletal infections including osteomyelitis, septic arthritis, and discitis.

Gastroenteritis is generally self-limiting, and symptoms typically include fever, abdominal pain, diarrhea, and nausea. Reactive arthritis which can involve both minor and major joints is usually seen 1 to 3 weeks after the onset of gastroenteritis symptoms [2].

A comprehensive review of the literature revealed only five prior case reports of *Y. enterocolitica* causing prosthetic joint infection, which includes 2 cases after total hip arthroplasty (THA) and 3 cases after total knee arthroplasty (TKA) [2–6]. These have been reviewed in the discussion section.

## 2. Case Presentation

A 70-year-old female with multiple medical comorbidities, including hypertension, end-stage renal disease (ESRD), and multiple prosthetic joints including a left total knee arthroplasty (TKA), presented to the emergency room with one-week history of left knee pain, erythema, and swelling. Prior to developing the symptoms in her knee, she suffered from loose stools and abdominal pain that had resolved by the time the patient developed pain in her knee. The patient denied any recent trauma, any rashes, or recent travel.

Physical examination was pertinent for tachycardia with a heart rate of 147 beats per minute, respiratory rate of 25 breaths per minute, temperature of 100.5 F, and oxygen saturation of 100% on room air. Blood pressure was 101/59 mmHg. The left knee was red, hot, and swollen. The range of motion of the left knee was restricted. The rest of her physical examination was unremarkable.

**Figure 1:**
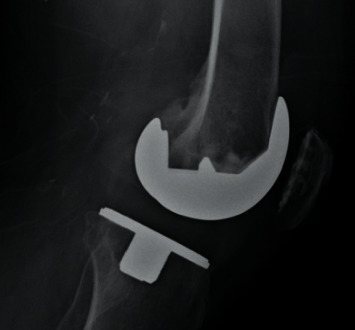
Lateral view of the left knee joint showing previous total knee replacement.

**Figure 2:**
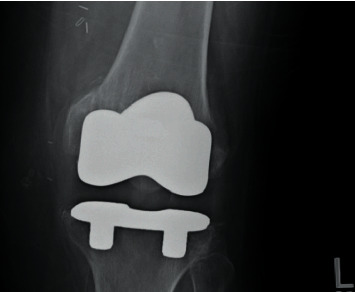
Anterior-posterior view of the left knee showing prior total knee replacement.

On laboratory work up, hemoglobin was 15.2 g/dL (13.5–17.5 g/dL); white blood cell (WBC) count, 14,900 cells/uL (4.5–11 k/uL), total bilirubin, 0.8 mg/dL (0.3–1.0 mg/dL); aspartate aminotransferase, 35 U/L (13–39 U/L); alanine aminotransferase, 33 U/L (4–33 U/L); alkaline phosphatase, 124 U/L (34–104 U/L); s. creatinine, 2.3 mg/dl; and sodium and potassium, within normal limits. Lateral and anterior-posterior view X-rays of the left knee joint showed prior total knee replacement but no other significant findings (Figures 1 and 2).

The initial differentials included septic arthritis of her prosthetic knee joint and reactive arthritis after her recent diarrhea. Gout and other rheumatologic conditions were less likely.

A preliminary diagnosis of sepsis due to septic arthritis was made, and supportive management with intravenous (IV) fluids, empiric antibiotics (vancomycin and zosyn), and antipyretics was initiated. Arthrocentesis was performed, and purulent fluid was drained. This was sent for Gram staining and cultures. Gram staining was significant for numerous WBCs and Gram-negative coccobacilli. The culture came back the next day growing Gram-positive coccobacilli that were subsequently identified as *Yersinia enterocolitis*. This was sensitive to all the antibiotics it was tested against. The blood cultures came back positive for *Yersinia enterocolitica* too. *Yersinia enterocolitica* was identified as the cause of the septic arthritis, which was likely the cause of her diarrhea prior to presenting.

The patient was seen by orthopedic surgery in consultation and underwent resection of her left TKA and placement of a biodegradable implant without any complications. The patient was also seen by infectious disease in consultation, and based on their recommendations, the patient was discharged home to complete 6 weeks of outpatient IV antibiotics in the form of 2 g of ceftriaxone daily considering her sepsis and prosthetic material in her knee joint. The patient completed this without any complications.

The patient was seen on follow-up, and her infection resolved completely. She has not had a recurrence or reinfection of her knee after over a year of follow-up.

## 3. Discussion

The majority (60%) of prosthetic joint infections are caused by Gram-positive cocci particularly *Staphylococcus aureus* and coagulase-negative staphylococci. Only 6–25% are caused by Gram-negative bacilli including *Pseudomonas aeruginosa*, *Escherichia coli*, and *Enterobacter* species [2]. Septic arthritis of a prosthetic joint caused by *Y. enterocolitica* is very rare. A review of the literature with the key words “*Yersinia enterocolitica* arthritis” and “*Yersinia enterocolitica* prosthetic joint” found only five cases of *Y. enterocolitica* prosthetic joint infections reported before. These are summarized in Table 1.

A review of the cases show that the age distribution of the patients ranged from 72 to 90 years. Three of them were female, and two were male patients. Most of them had some comorbidities, with diabetes being the most common. Three of the previously reported cases were reported after total knee arthroplasty (TKA), and two were reported after total hip arthroplasty (THA) [2–6]. The time duration between the placement of the prosthesis and the infection ranged from 1 year to 15 years. None of the cases had associated enterocolitis reported. Culture of the joint debridement tissue was positive for *Y. enterocolitica* in all the cases. Only one case was associated with positive blood cultures of *Y. enterocolitica*.

Generally, the pathogenic serotype of *Y. enterocolitica* produces beta lactamases and is found to be resistant to penicillin, ampicillin, macrolides, and many first-generation cephalosporins [2]. *Y. enterocolitica* prosthetic joint infection cases were mainly treated with aminoglycoside, third-generation cephalosporins, and fluoroquinolones (Table 1). Aminoglycosides have higher adverse effects including nephrotoxicity, ototoxicity, and neuromuscular blockade and therefore should be used only in patients who are unable to tolerate later-generation cephalosporins or fluoroquinolones. No controlled trials have been done which can guide the total duration of treatment for *Y. enterocolitica* extraintestinal infections, but current recommendations range between 2 and 6 weeks of total antibiotic duration [2]. Our patient received a total of six weeks of IV antibiotics and made a successful recovery.

One case was described by Hougaard et al. after THA, where the prosthetic joint was replaced and broad-spectrum antibiotics were used [2]. In contrast, all other cases were successfully treated with antibiotics without the need of revision arthroplasty [1, 4–6]. The usual treatment for a prosthetic joint infection that is hematogenous in origin would be debridement antibiotics and implant retention (DAIR). But, in our case, the patient was immunocompromised due to uncontrolled diabetes and end-stage renal disease for which she was on dialysis. As such, the surgery and infectious disease team thought that she would not be a good candidate for DAIR treatment due to her immunosuppression. Once the patient was treated for the infection and it resolved, she underwent a repeat total knee arthroplasty a few months later.

It is important to differentiate *Y. enterocolitica* septic arthritis from reactive arthritis which is a common sequela of *Y. enterocolitica* infection. Reactive arthritis is usually associated with HLA-B27 and presents one to three weeks after acute enteritis with sterile joint arthritis, enthesitis, and dactylitis [7]. In our case, HLA-B27 was negative. Also, the onset of joint pain was within one week of enteritis. These suggested septic arthritis as a more likely etiology, and this was confirmed by the positive blood and synovial fluid culture for *Y. enterocolitica*. Synovial fluid is sterile in septic arthritis which is a major differentiating point from septic arthritis.

There are a few limitations in our case study and associated literature review. No stool culture was done for our patient when she initially had diarrhea. We presumed the septic arthritis was most likely from a gastrointestinal source considering the recent diarrhea. In addition to this, the patients reviewed in our study were treated with different antibiotics and in many cases, the duration of treatment was not clearly mentioned. As such, our study may not be generalizable to all patients with similar clinical presentations and a decision on how to treat should be made on based on the patient's clinical history.

## Figures and Tables

**Table 1:** All the prior reported cases of *Yersinia enterocolitica* prosthetic joint septic arthritis.

Case	Patient age/sex	Time since prosthesis placed	Prosthetic joint	Comorbidities	Associated colitis/sepsis	Culture positive?	Debridement of joint/prothesis removal	Antibiotics	Outcome	Recurrence
Hougaard (1990)	72	10 years	THA	—	-/-	Yes, O3:4	Yes	Cloxacillin/gentamicin beads/cefotaxime/piperacillin/gentamicin/mecillinam/ciprofloxacin	Recovered	None
Oni JA (1991)	84	7 years	TKA	—	-/+	Yes, O9	No	Cefuroxime-axetil	Recovered	None
Maeda (2018)	62	1 year	THA	DM, HTN, HLD, Afib	-/-	Yes	No	Ceftriaxone, ciprofloxacin	Recovered	None
Iglesias (2002)	80	10 years	TKA	DM, mechanical aortic valve	-/-	Yes	No	Co-trimoxazole, ciprofloxacin	Recovered	None
Jalava-Karvinen (2013)	90/M	15 years	TKA	HTN, CAD	-/-	Yes	No	Ceftriaxone, piperacillin + tazobactam, ciprofloxacin, meropenem	Recovered	None
Our case	70/F	3 years	TKA	HTN, ESRD	+/+	Yes	Yes	Ceftriaxone *×* 6 weeks	Recovered	None
